# Effects of Photoperiod on Epididymal and Sperm Morphology in a Wild Rodent, the Viscacha (*Lagostomus maximus maximus*)

**DOI:** 10.5402/2013/128921

**Published:** 2012-12-02

**Authors:** A. M. Cruceño, J. C. de Rosas, M. Fóscolo, E. M. Chaves, L. Scardapane, S. Dominguez, C. Aguilera-Merlo

**Affiliations:** ^1^Cátedra de Histología, Facultad de Química, Bioquímica y Farmacia, Universidad Nacional de San Luis, Avenida Ejército de Los Andes 950-No. 2 Piso, 5700 San Luis, Argentina; ^2^Instituto de Histología y Embriología, Facultad de Ciencias Médicas, Universidad Nacional de Cuyo CONICET, Mendoza 5500, Argentina

## Abstract

The viscacha (*Lagostomus maximus maximus*) is a seasonal South American wild rodent. The adult males exhibit an annual reproductive cycle with periods of maximum and minimum gonadal activity. Four segments have been identified in the epididymis of this species: initial, caput, corpus, and cauda. The main objective of this work was to relate the seasonal morphological changes observed in the epididymal duct with the data from epididymal sperm during periods of activity and gonadal regression using light and scanning electron microscopy (SEM). Under light and electron microscopy, epididymal corpus and cauda showed marked seasonal variations in structural parameters and in the distribution of different cellular populations of epithelium. Initial and caput segments showed mild morphological variations between the two periods. Changes in epididymal sperm morphology were observed in the periods analyzed and an increased number of abnormal gametes were found during the regression period. During this period, anomalies were found mainly in the head, midpiece, and neck, while in the activity period, defects were found only in the head. Our results confirm that the morphological characteristics of the epididymal segments, as well as sperm morphology, undergo significant changes during the reproductive cycle of *Lagostomus*.

## 1. Introduction

Animal species that develop their life cycle in contact with nature must adapt physiologically to the annual changes conditioned by the environment. The role of environment signals in synchronization has been described in previous reports, especially the circannual regulation of pituitary and gonadal function by photoperiod [1, 2]. Depending on the season, adult males have reproductive cycles that are expressed by morphological and biochemical changes in their reproductive organs [3]. Also, some species show testes devoid of all signs of spermatogenic activity during the winter months [4]. 

Moreover, mammalian spermatozoa produced in the testis must mature in the epididymis to acquire their fertilizing capability [5, 6]. Specific proteins secreted by epithelial cells of the epididymis and factors generated by accessory glands modulate the structure and function of the sperm plasma membrane. The size and secretory activity of epithelia in the epididymis as well as in the accessory glands are influenced by testosterone [7].

In this study, the model animal used is the viscacha (*Lagostomus maximus maximus*), a South American rodent which lives in burrows built deep in the ground and only leaves its cave at dawn to look for food [8, 9]. Studies carried out in our laboratory have demonstrated that this rodent has seasonal reproduction, the environmental photoperiod being the main synchronizer of the reproductive cycle along the year. In its habitat, the adult male exhibits a reproductive cycle characterized by three periods. The period of highest reproductive activity may be observed during long summer days and early autumn and is followed by a short period of gonadal regression during winter. The reproductive system gradually recovers during spring to reach its highest activity in summer again [10–17]. 

Based on previous studies in viscacha and due to its morphological and functional complexity, the epididymis has been divided into four segments: initial, caput, corpus, and cauda [18], in agreement with Serre and Robaire [19] in rat epididymis. The histological characteristics of the different segments of viscacha epididymis show a pseudostratified epithelium constituted by several cellular types: principal, basal, clear, halo, narrow, and apical cells. The distribution of epithelial cell populations varies in the different epididymal segments. Principal and basal cells are the most frequent types found along the total length of the duct. Clear cells are not present in the initial segment but are located in the other segments. Halo cells are observed throughout the epididymis. Both narrow and apical cells are present only in the initial segment [18, 20]. Studies in rats [20, 21] and hamster [22, 23] have reported similarities to viscacha, in terms of epithelial cell types of the epididymis. 

However, studies linking seasonal morphological changes of epididymal with epididymal sperm morphology are still unknown.

The main objectives of this work were to characterize quantity and quality of epididymal spermatozoa and relate the seasonal morphological changes observed in the epididymal duct during periods of activity and gonadal regression using light and scanning electron microscopy.

## 2. Materials and Methods

### 2.1. Animals

A total of six adult viscachas weighing 4–8 Kg were captured in their habitat near San Luis, Argentina (33°20′ south latitude, 760 m altitude) during the periods of maximum gonadal activity (summer–autumn) and gonadal regression (winter). The animals were anesthetized intramuscularly with a combination of ketamine and xylazine at a dose of 12 mg/kg and 0.4 mg/kg, respectively, and sacrificed. In San Luis, summer days have 14 h of light with an average temperature of 25°C. In winter, the light phase decreases to 10 h and the average temperature is 10°C. The average rainfall is 400 mm in summer and 18 mm in winter (National Meteorological Service, http://www.smn.gov.ar/). Appropriate procedures were performed to minimize the number of animals used and suffering. The experimental design was approved by the Local Ethics Committee and was in agreement with the guidelines of the National Institute of Health (NIH, USA) for the use of experimental animals. Moreover, the Biodiversity Control Area of the Ministry of the Environment of San Luis (Argentina) approved a study protocol to carry out scientific research within the territory of this province (resolution no. 03 PRN-2011).

### 2.2. Morphometric Study

Five animals from each period of reproduction cycle were studied. For each animal, 15 sections for each epididymal segment were randomly chosen. Measures were made in plastic sections stained with toluidine blue (TB) using an ocular micrometer. The following measures were taken in transverse epididymal sections: luminal diameter using an objective of 10X; height of the epithelium (excluding the stereocilia), thickness of lamina propria (both with the 40X objective), length of the stereocilia using an immersion objective to 100X. Data were finally expressed in micrometers.

### 2.3. Scanning Procedure

 Fragments of the initial segment, caput, corpus, and cauda segments of the epididymis were obtained from adult males of viscacha. The tissue samples were fixed by immersion in a formaldehyde-glutaraldehyde mixture in 0.1 M sodium-phosphate buffer at pH 7.2, 4°C, and 24 h. These fragments were washed in the buffer, post-fixed in 1% osmium tetroxide in 0.1 M sodium-phosphate buffer, and immersed in 2% tannic acid for 90 minutes [24]. The tissues were dehydrated with ethanol and immersed in uranyl acetate. Finally, they were dried with liquid carbon dioxide. The specimens were observed with a Philips SEM-515 microscope at 20 kV after sputter coating with gold.

### 2.4. Spermatozoa Examination

Spermatozoa were collected by mincing the cauda epididymis in 2.0 mL of HECM-3 medium, and epididymal tissue was removed by centrifugation [25]. Sperm concentration was estimated by hemocytometer. Spermatozoa samples were divided into two categories, normal and abnormal, according to the strict sperm morphology criteria. The morphological abnormalities were divided into three categories: head, midpiece, and neck and tail defects. The morphological abnormalities can be found in Table 2. 

Sperm were photographed with a digital camera incorporated into an optical microscope (×400 or ×1000) and measured with an image analysis software. We determinate head width, head, midpiece, and total length.

### 2.5. Statistical Analysis

Means and standard errors were calculated for all data sets. Differences between groups were evaluated using one-way analysis of variance (ANOVA) or Kruskal-Wallis test (nonparametric ANOVA) followed by pairwise comparisons using a Tukey-Kramer or Dunn test, respectively. A *P* value < 0.05 was accepted as statistically significant.

## 3. Results

 In previous observations by light microscopy, we demonstrated that the epididymis of viscacha had four regions: initial segment, caput, corpus, and cauda. All regions are lined by a pseudostratified cylindrical epithelium with stereocilia. Below the epithelial basement membrane is located a thin lamina propria and smooth muscle fibers. The muscle layer of the epididymal duct is thin in most parts of the epididymis, but thickness increased markedly in the region of the cauda epididymis. 

### 3.1. Initial Segment (IS) and Caput

The analysis of these parameters in the four segments of the epididymis corresponding to both periods is shown in Table 1. In the IS, the luminal diameter is reduced and the thickness of the lamina propria is increased during the regression period contrary to what happened during the period of gonadal activity. In the caput, none of parameters showed significant changes along the annual reproductive cycle. 

**Figure 1:**
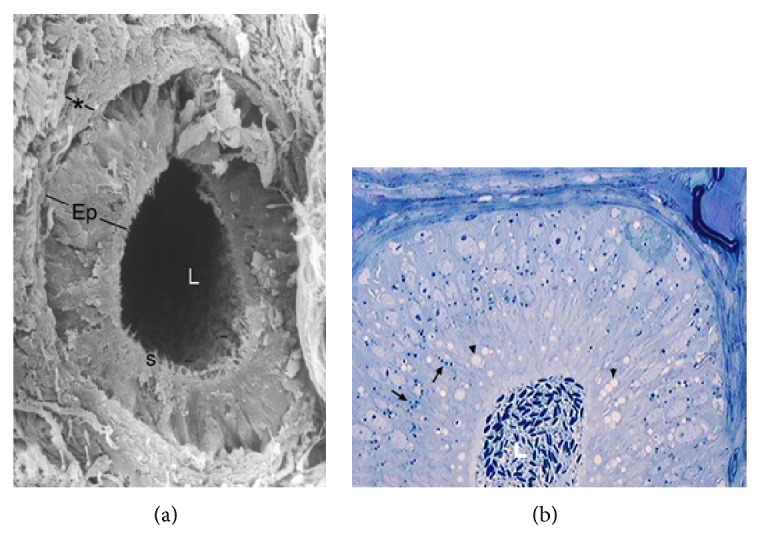
Epididymal segments in the active period. (a) Image showing the organization of a section of initial segment obtained by scanning electron microscope. Note the greater height of the epithelium (Ep) and the thickness of the lamina propria (asterisk) below the epithelium. L: lumen; s: stereocilia. SEM: ×600. (b) Semithin cut of a section of epididymal caput emphasizing the presence of abundant lipid droplets (arrowhead) and dense bodies (arrow) in the cytoplasm of principal cells. Numerous sperm are stored in the epididymal lumen (L). TB: ×400.

**Figure 2:**
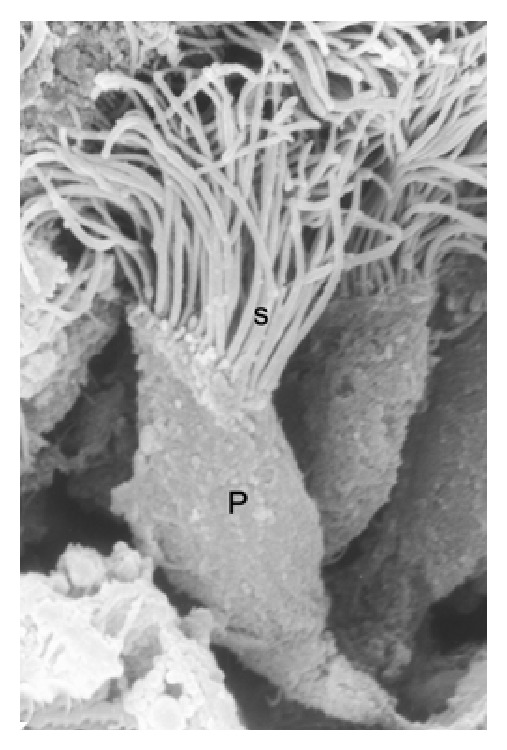
Principal cell (P) in the epididymal epithelium. Image observed to scanning electron micrograph showing the morphology of this cell. Note the presence of extensive stereocilia (s) on the apical surface of this cell. SEM: ×7000.

**Figure 3:**
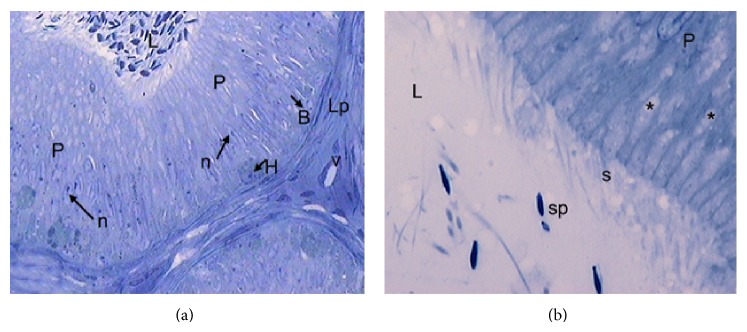
Epididymal segments in the gonadal regression period. (a) Semithin cut of a section of epididymal caput showing principal cells (P) with low cellular activity. Their nuclei (n and arrow) are elongated and have scarce cytoplasmic content. B: basal cell (arrow); H: halo cell (arrow); Lp: lamina propria; v: blood vessel; L: lumen. TB: ×400. (b) Note the clustered arrangement of the stereocilia (s) of principal cells (P) and the poor presence of sperm (sp) stored in this segment. Asterisks: lipid droplets. L: lumen. TB: ×1000.

During the period of gonadal activity, these segments showed high epithelium, with principal cells as the predominant type (Figures 1(a) and 1(b)). These cells have an elongated nucleus perpendicular to the lamina propria and in the apical surface are located stereocilia long and thin (Figure 2). In semithin sections it can be observed that the principal and halo cells show great development of their cellular components, which indicates intense cellular activity during this period. Numerous structures similar to lipid cells and dense bodies were observed in the cytoplasm of the principal cells. An important number of spermatozoa are in contact with long stereocilia of these cells (Figures 1(b)–3(a)). 

In the regression period, few histological changes are observed in the IS, while in the caput of the epididymis, several epithelial cells are in process of desquamation. Most principal cells exhibit nuclei with irregular edges and in their cytoplasm are observed structures similar to lipid droplets. Stereocilia are organized in groups and contain scarce anchored spermatozoa (Figure 3(b)). 

### 3.2. Corpus

In this segment, we have not observed significant differences in the structural parameters along the reproductive cycle (Table 1). 

**Figure 4:**
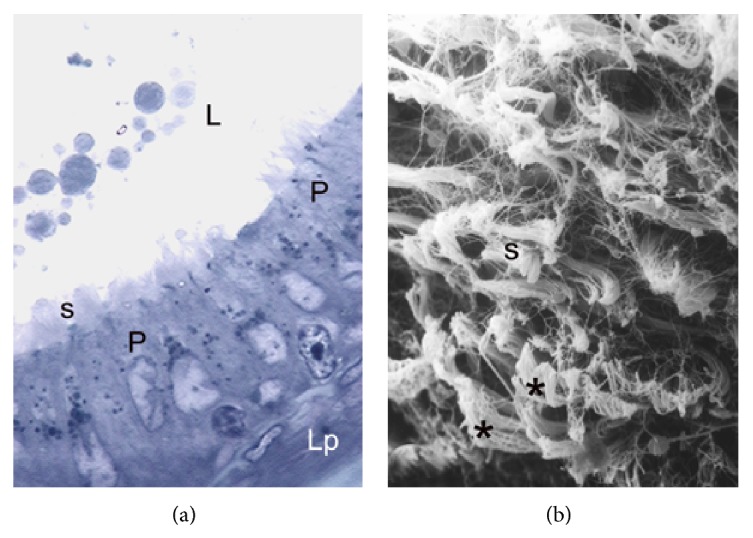
Epididymal corpus in active period. (a) Semithin cut showing principal cells (P) with characteristics of high cellular activity. Observe the length and arrangement of the stereocilia (s). L: lumen. Lp: lamina propria. TB: ×1000. (b) Scanning electron micrograph showing in the epididymal corpus an increased secretion (asterisk) of epididymal epithelium into the lumen. Note the clustered arrangement of the stereocilia (s) during the period of greatest reproductive activity. SEM: ×1200.

During the active period, the principal cells of this segment exhibit characteristics typical of highly active cells (Figure 4(a)). They are tall, with lax nucleus and irregular edges. The principal cells present a wide exposition area towards the lumen of the duct and stereocilia of lower length than those of the previously described segments. In the lumen of this segment, abundant content of secretory material and cells detached from the epithelium lining are observed (Figure 4(b)). Also, a poorly developed lamina propria is observed below the epididymal epithelium. 

**Figure 5:**
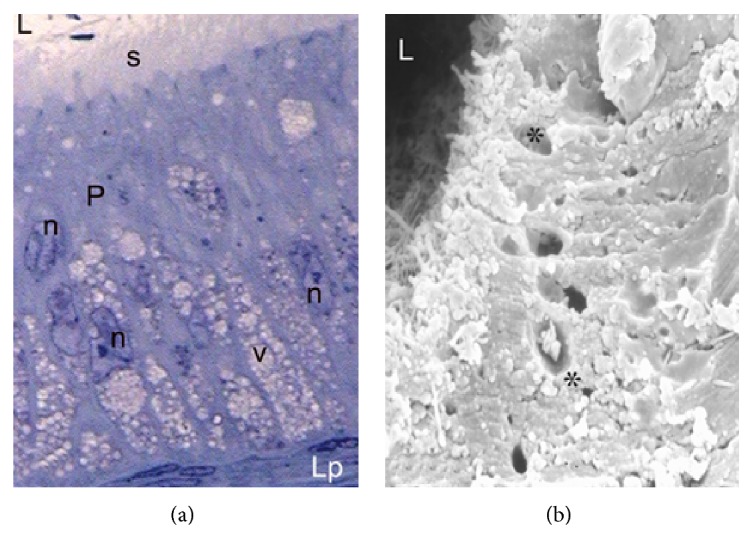
Epididymal corpus in gonadal regression period. (a) Semithin cut showing irregular nuclei (n) and abundant cytoplasmic vacuoles (v) in principal cells (P). Note the greater height of the epithelium in relation to that observed in the active period. L: lumen; s: stereocilia; Lp: lamina propria. TB: ×1000. (b) Scanning electron micrograph showing holes or depressions (asterisks) in the cytoplasmic surface of epithelial cells caused by marked epithelial vacuolation. L: lumen. SEM: ×3500.

In the regression period, the principal cells exhibited nuclei with irregular edges, and the cytoplasm shows abundant vacuoles of irregular sizes and shapes, sometimes fused with each other (Figure 5(a)). The lumen and lamina propria show characteristics similar to those described for the active period. Under the scanning electronic microscope, a wider extension of principal cells with lengthened cellular apex are observed. The cytoplasmic surface exhibits depressions or holes that can be related with the large vacuoles present in their cytoplasm (Figure 5(b)). 

### 3.3. Cauda

In this segment is observed a decrease in the luminal diameter during the regression. On the other hand, the epithelium height and the thickness of lamina propria are increased during the regression (Table 1). 

**Figure 6:**
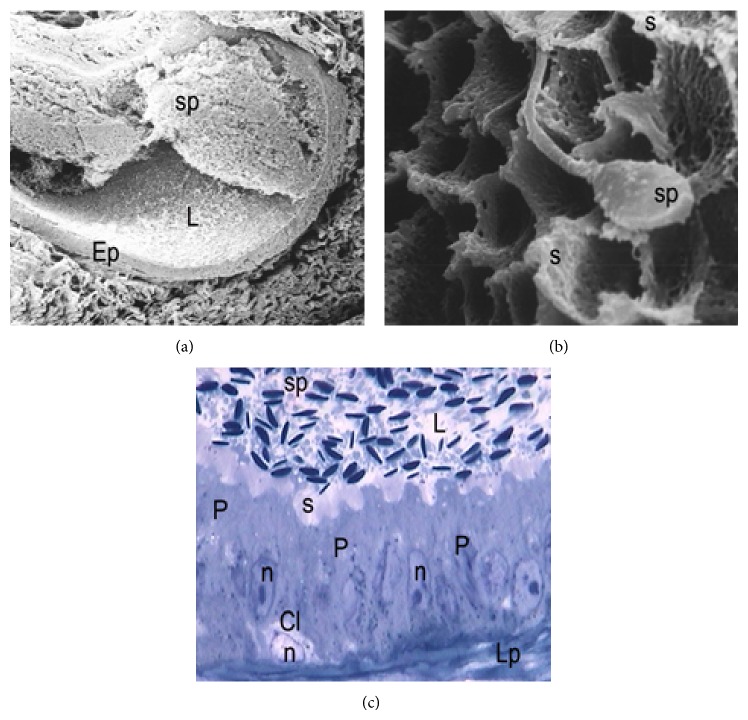
Epididymal cauda in active period. (a) Epididymal section observed at low magnification by scanning electron microscopy showing a low height of the epithelium (Ep), and a dilated lumen (L) with abundant sperm (sp). SEM: ×150. (b) In this image at higher magnification shows the organization of the stereocilia (s) forming sperm (sp) reservoirs. SEM: ×7000. (c) Semithin cut obtained from the same region of the epididymal cauda. A high number of sperm (sp) are stored in the epididymal lumen (L). Cl: clear cell; P: principal cells; n: nuclei; Lp: lamina propria; s: stereocilia. TB: ×1000.

 In the active period, the epididymal sections cut transversally and observed at low magnification under the scanning electronic microscope show a low epithelium and a wide lumen totally occupied by a dense sperm mass (Figure 6(a)). Details at greater magnification revealed the organization of the stereocilia in the distal region of the cauda segment, forming true sperm reservoirs (Figure 6(b)). The epithelium exhibited cells with lax nucleus and short stereocilia which are grouped forming a low brush border. A lamina propria was observed surrounding the duct epithelium, more developed than in the previous segments of the active epididymis (Figure 6(c)). 

**Figure 7:**
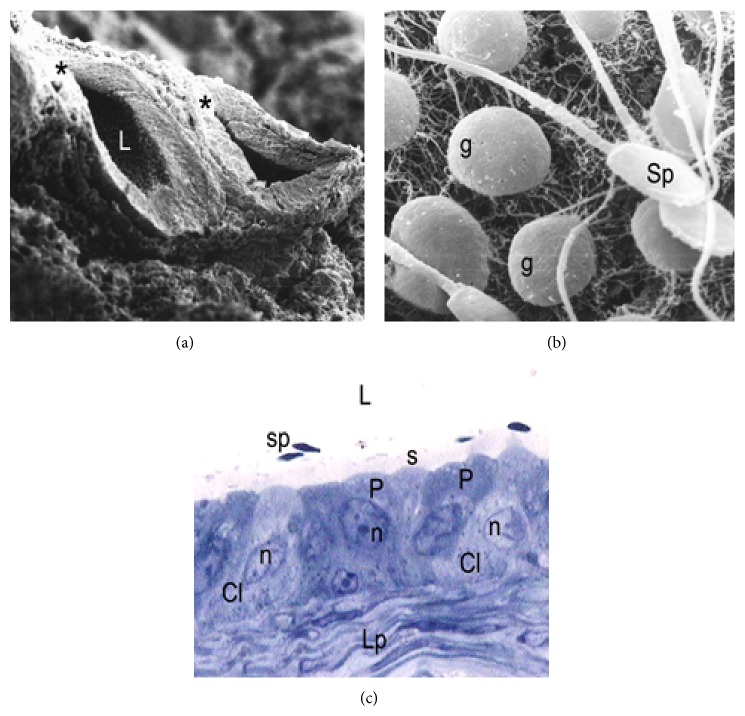
Scanning electron micrograph of the epididymal cauda during the period of regression. (a) Low magnification image showing epididymal sections (asterisk) with reduced luminal diameter in relation to the observed during the active period. L: lumen. SEM: ×300. (b) In the epididymal lumen at higher magnification is observed low sperm content (sp) and abundant germ cells (g) detached. SEM: ×6000. (c) Semithin cut obtained in the same region of the epididymal cauda showing principal cells (P) and numerous clear cells (Cl) with lax and irregular nuclei (n). Note the low content of sperm (sp) in the lumen (L) and increased thickness of the lamina propria (Lp), in relation to the observed during the active period. s stereocilia. TB: ×1000.

 During the regression period, the epididymal cauda exhibits important morphological changes as compared to the active period. SEM images revealed a significant reduction of the luminal diameter (Figure 7(a)). The lumen exhibits lower presence of stored sperm cells and abundant detached epithelial cells of testicular and epididymal origin (Figure 7(b)). In this period, a higher presence of clear cells was observed in the cauda epididymal epithelium as compared to the active period (Figure 7(c)). 

**Figure 8:**
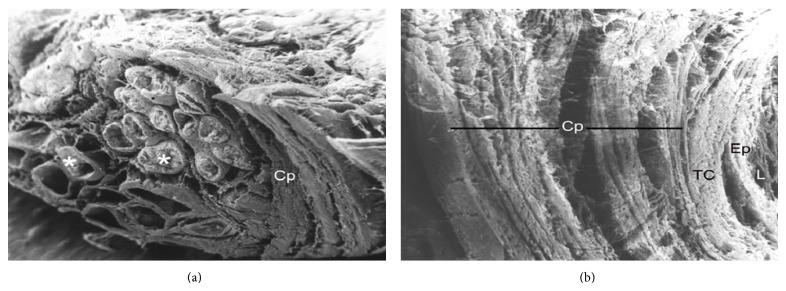
Epididymal capsule. (a) Image obtained by scanning electron microscope in the distal region of the epididymal cauda showing numerous sections (asterisks) surrounded by a thick capsule (Cp). SEM: ×50. (b) Note the concentric organization of the connective layers forming the capsule (Cp) of the epididymis. Ep: epithelium; connective tissue; L: lumen. SEM: ×200.

 A particular feature observed in the viscacha epididymis is that the cauda segment is wrapped by a thick fibro-muscular capsule formed by several concentric layers of connective and muscular tissue. This capsular structure maintains the same histoarchitecture throughout the reproductive cycle of *Lagostomus*. (Figures 8(a)–8(b)). 

### 3.4. Normal Morphology of Spermatozoa

**Figure 9:**
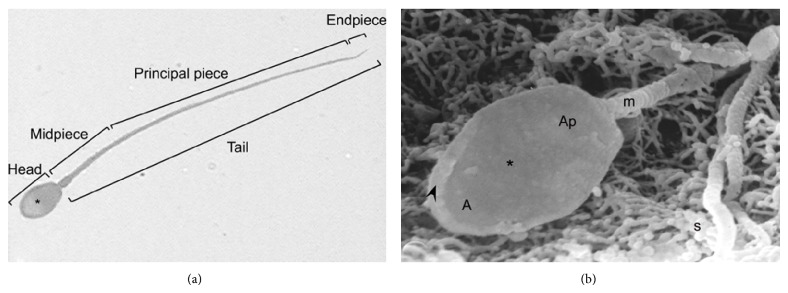
Normal sperm morphology of viscacha. (a) Sperm obtained from the epididymal fluid and observed by light microscopy. Bright green staining. Asterisk: equatorial segment. ×1000. (b) Image obtained by scanning electron microscope showing a normal sperm located inside of the epididymis and anchored to the stereocilia (s) of the epithelium. Note the apical thickening (arrowhead) of the sperm head and the mitochondrial sheath (m) placed in the middle part of the flagellum. In the sperm head acrosomal (A) and postacrosomal (Ap) regions are identified. Asterisk: equatorial segment. SEM: ×10.000.

The normal morphology of the spermatozoon is shown in Figure 9(a). The head isflattenedand scoop-shaped, withan average length of 6.25 ± 0.10 *μ*m and a width of 4.2 ± 0.03 *μ*m. The acrosomal region, covering the anterior third of the head, is separated from the postacrosomal region by the presence of an equatorial segment visible under light microscopy. The total length of the head is 6.25 ± 0.1 *μ*m and the total length of the flagellum varies from 43.8 ± 0.93 to 48.67 ± 0.42 *μ*m. Ultrastructural studies under SEM permitted to distinguish an acrosomal and a postacrosomal region separated by the equatorial segment. A thickened mitochondrial sheath can be seen in the midpiece of the flagellum (Figure 9(b)). 

### 3.5. Sperm Morphology in the Different Periods of Reproductive Cycle

The abnormal morphologies observed during the active and regression periods are shown in Table 2. The percentage of the abnormal and normal sperm examined shows statistically significant differences between the periods analyzed. 

**Figure 10:**
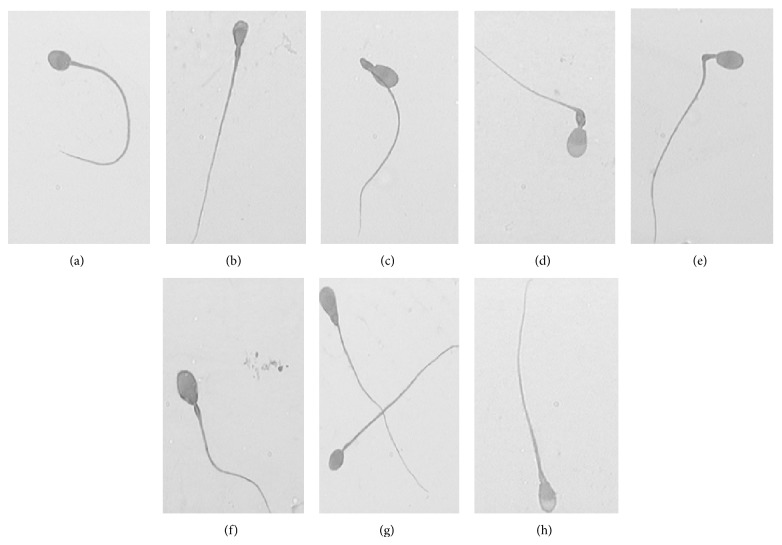
Abnormal sperm morphology of viscacha. Image obtained from the extended sperm epididymal fluid and observed by light microscopy. Bright green staining. ×1000. (a) Round head. (b) pyriform head. (c) Bent tail. (d) Thick insertion. (e) Bent neck. (f) Amorphous. (g) Amorphous and round head. (h) Tapered.

During the active period, the study of sperm morphology by light microscopy showed a low percentage of abnormal spermatozoa. The main defects found were conical head (2.74 ± 0.06) and pyriform head (1.89 ± 0.03). Minor defects such as round head (0.86 ± 0.01), bent tail (0.90 ± 0.02), or coiled tail (0.93 ± 0.01) were also observed. In the period of regression, significant variations in the percentage of abnormal spermatozoa compared to active period were observed. The main defects were located in the head with round shapes (6.51 ± 0.06) or tapered (4.43 ± 0.04), amorphous (5.2 ± 0.05) or asymmetric (4.51 ± 0.04), and thick necks (9.63 ± 0.03). Minor defects were detected as pyriform heads (0.77 ± 0.02) and coiled tails (1.48 ± 0.02). The most important morphological abnormalities are shown in Figures 10(a)–10(h). 

## 4. Discussion

 Wild mammals with seasonal reproduction are able to respond to environmental factors through physiological and behavioral changes in anticipation of the arrival of a new season. The switching on and off of reproductive function during the annual breeding cycle is the most striking example of such a photoperiodically induced process [11, 26]. 

 In the present study, the results showed marked morphological changes in all segments of the epididymis of viscacha between the reproductive periods analyzed. All epididymal segments show structural variations and changes in the distribution of different cell populations of epididymal epithelium during the reproductive cycle of viscacha. In addition, morphometric analysis of luminal diameter and epithelial height and thickness of the lamina propria of the rodent epididymis allowed us to verify that these parameters vary significantly only in the epididymal cauda. During the short period of gonadal regression, the cauda segment has a smaller lumen diameter, decreased sperm population, and increased thickness of the lamina propria. 

 In active epididymis of viscacha, the apical surface of principal cells show a reduction in the length of stereocilia in the cauda. Some marsupials [27, 28] and domestic animals [29] have been observed features similar to those observed in viscacha, where short stereocilia are replaced by the low brush border of microvilli. Probably, the functional implication of the brush border is that it favors the endocytosis and exocytosis processes, which occur simultaneously between the epithelial cells and the fluid of the luminal content, as suggested by suggested by Arrighi et al. [30] for efferent ductules of domestic equidae and Schimming [29] in epididymis of dog.

The rates of abnormal sperm morphology were statistically different between the two periods analyzed. In the regression period, the quantitative results indicate that the number of abnormal spermatozoa increased progressively and the defects are located more frequently in the head and neck of the sperm. In testes, from the regression period of this same species, Muñoz et al. [15] observed reduced seminiferous tubules, epithelial disorganization and vacuolization, lower number of germ cells, specifically spermatids, and cells in process of degeneration. Furthermore, Sertoli cells exhibit seasonal variations of the nuclear and cytoplasmic features [31]. In epididymis of viscacha, Aguilera-Merlo et al. [18] detected significant reduction in the number of all cell populations corresponding to the cauda segment and a decrease in luminal content. On the other hand, studies conducted on binding assays between heterologous gametes of hamster oocytes versus epididymal spermatozoa of viscacha clearly show a high gamete interaction between viscacha spermatozoa and hamster oocyte with and without zona pellucida during the period of maximum gonadal activity, while this capacity of primary binding between gametes diminishes significantly in the period of testicular and epididymal regression [32]. According to the results obtained in this work, the observed increase of defects in the neck and middle part of the epididymal spermatozoa of viscacha, without capacitation, is probably due to a failure of the testicular spermatogenesis or a decrease in the ability of abnormal spermatozoa reabsorption by the epididymal epithelium during the maturation process. These results allow us to postulate that a higher number of sperm with abnormal morphology correlate with an impaired ability to perform a primary heterologous binding during the regression period of viscacha.

In conclusion, these results confirm that the reproductive activity of *Lagostomus* is cyclic as a function of the annual seasonal periods that are influenced by environmental conditions and regulated for hormonal changes observed in the serum testosterone levels. On the other hand, each segment of the epididymis probably performs specific functions in the capacitation and maturation of spermatozoa and shows a specific epithelial cytoarchitecture likely conditioned by the histophysiology of the preceding segment.

Data for semen quantity and quality in relation to the seasonal changes in morphometry and morphology of the epididymis in viscacha may contribute to achieve a better understanding of epididymal physiology in the seasonal reproduction of this rodent. 

## Figures and Tables

**Table 1:** Means values ± SEM of structural parameters in each epididymal segment during periods of activity and regression.

Segment	Parameters	Period		
		Active	Regression	*P*
Initial	Luminal diameter	128 ± 4.7	84 ± 2.3	<0.001^*^
	Epithelium height	57.3 ± 1.3	48 ± 4.6	>0.05
	Thickness of lamina propria	7.6 ± 2.3	9.6 ± 1.3	<0.05^*^
	Stereocilia length	7.3 ± 0.6	6.6 ± 0.6	>0.05

Caput	Luminal diameter	117.3 ± 1.3	105.3 ± 1.3	>0.05
	Epithelium height	53.3 ± 3.5	45.3 ± 1.3	>0.05
	Thickness of lamina propria	6.7 ± 1.3	8.0 ± 2.3	>0.05
	Stereocilia length	4.3 ± 0.7	6.3 ± 0.7	>0.05

Corpus	Luminal diameter	148 ± 2.3	136 ± 4.6	>0.05
	Epithelium height	36 ± 2.3	44 ± 2.3	>0.05
	Thickness of lamina propria	8 ± 2.3	9.3 ± 1.3	>0.05
	Stereocilia length	5.3 ± 0.6	5.6 ± 0.6	>0.05

Cauda	Luminal diameter	443 ± 1.9	106.7 ± 1.3	<0.001^*^
	Epithelium height	10.6 ± 1.3	21.3 ± 1.3	<0.05^*^
	Thickness of lamina propria	8.3 ± 2.3	16.4 ± 2.3	<0.05^*^
	Stereocilia length	5.6 ± 0.3	5.3 ± 0.3	>0.05

^*^Values with significant difference in regression period.

**Table 2:** Abnormal morphology rates (%) of viscacha spermatozoa during periods of activity and regression.

	Activity	Regression	
Number of samples	5	5	
Sperm parameters			

Concentration	485 ± 13.5 × 10^6^	72 ± 8.5 × 10^6^	

Morphology			*P *

Normal	93.2 ± 0.46	65.2 ± 0.32	<0.001
Abnormal	7.8 ± 0.004	33.8 ± 0.004	<0.001
*Head defects *			
Pyriform	1.96 ± 0.004	0.75 ± 0.004	<0.001
Round	0.98 ± 0.003	6.76 ± 0.003	<0.001
Tapered	2.94 ± 0.004	4.51 ± 0.004	<0.001
Amorphous	—	5.26 ± 0.01	
*Neck and midpiece defects *			
Asymmetrical	—	4.51 ± 0.004	
Thick insertion	—	9.77 ± 0.008	
*Tail defects *			
Bent	0.98 ± 0.004	—	
Coiled	0.98 ± 0.004	1.5 ± 0.01	<0.001
*Excess residual cytoplasm *			
>one-third head	—	0.75 ± 0.005	

The values are expressed as mean ± SEM. The significant differences were determinated by student's *t*-test.
